# Treating Alzheimer’s Disease (AD) with Light and Sound

**Published:** 2020-02-12

**Authors:** Walter J Lukiw

**Affiliations:** Department of Neurology, Louisiana State University Health Sciences Center, USA

## Commentary

Normal human brain functions through the complex acquisition, coordination, integration and processing of multiple and different afferent inputs and amongst the most prominent and important of these are the environmentally/externally-sourced input of light and sound. Together visual and acoustic stimulation accounts for about ~85% of the afferent input to the human brain. The natural generation of gamma oscillations in the brain - between 25 Hz and 140 Hz from this coordinated and integrated neural activity are thought to underlie multiple brain functions as diverse as attention, behavior, cognitive processing and memory. These natural oscillations, sometimes referred to as biological rhythms or biorhythms, may be significantly disrupted as we age and especially so in insidious, age-related, progressive and ultimately lethal neurological disorders such as Alzheimer’s Disease (AD) and frontotemporal dementia (FTD). The recent research work by Li-Huei Tsai’s research group at MIT on combined acoustic and visual gamma oscillation entrainment and its significantly positive effects on the cognitive functions of neural cell networks raises the intriguing possibility that artificial visual and acoustic stimulation of the brain can be successfully exploited to preserve both neural connectivity, neuronal densities and synaptic signalling function and elicit significant neuroprotection against progressive, age-related inflammatory and degenerative neuropathology [1].

The first reports of full-spectrum light- and acoustic-therapy-white light and white noise for the clinical treatment of anxiety, depression, seasonal-affective and related mood disorders over ~40 years ago underscore the tremendous impact that visual-and-acoustic stimulation has on global brain function, neurochemistry, signal integration, circadian rhythm, cognitive processing, biorhythms and behavior [2–8]. The Adaikkan et al. paper significantly extends our understanding of the brain mechanisms involved, including the potential therapeutic applications involving the calculated application of light and sound [1]. Initially, these investigators used a LED-flickering device in the application of a patterned visual stimulation combined with an acoustic component in a multisensory paradigm in an amyloid over-expressing, cognition-compromised transgenic murine model of AD (TgAD, the 5xFAD model) that involves, in part, the entrainment of gamma oscillations at 40-Hz *via* the visual circuitry. This novel treatment, referred to as the ***‘gamma entrainment using sensory stimuli’*** (GENUS) procedure, resulted in multiple unexpected neurological benefits including (i) the preservation of neuronal and synaptic density across multiple brain regions, (ii) amyloid and end-stage lipoprotein clearance, (iii) a reduced inflammatory response from microglia and (iv) enhanced cognition and cognitive performance [1,3,6,9].

Indeed, utilizing GENUS in the well characterized amyloid-beta (Aβ) peptide-overproducing 5xFAD TgAD murine model, this novel technique was further shown to impart a significant number of global neuroprotective and beneficial effects, including the amelioration of several key AD-characteristic neuropathological deficits that include: (i) a decrease in neuroinflammatory biomarkers, (ii) a significant decrease in both neuronal and synaptic loss, (iii) a significant decrease in tau phosphorylation (tau hyperphosphorylation is an important precursor to the formation of neurofibrillary tangles) and (iv) perhaps most importantly, a significant reduction in amyloid plaque load and plaque-associated neuropathology.

The use of conventional or ‘novel’ pharmacological and immunological approaches in the literature is endemic with reports of repeated failures in AD clinical trials that target the neuropathological hallmarks of the AD process: amyloidogenesis, ApoE anomalies, tau-based hyperphosphorylation and neurofibrillary tangle formation, defective neurotropism, pro-inflammatory neuropathology and neuronal and synaptic atrophy and decline. Importantly, the implementation of a non-invasive stimulation procedure for modulating oscillatory stimulation in the brains of aged adults should contribute to the foundation for the advancement of more efficacious, non-pharmacological interventions that directly target the highly integrated and interdependent mechanisms of brain circuitry. These include multiple and highly integrated aspects of amyloidogenesis, the clearance of age-related lesions, including senile plaques and neurofibrillary tangles from the brain, runaway brain inflammation, behavioral, perceptive and memory deficits and cognitive decline. Proven remarkably successful against several neurodegenerative phenotypes using the 5xFAD TgAD and other transgenic murine models, currently GENUS-based photobiomodulatory interventions are undergoing extensive clinical trials in the US and China in both AD and FTD patients. The manipulation of global neural-network oscillations using photobiomodulation with an acoustic component that significantly shift neural cells to a less degenerative, *‘neuroprotected’* state clearly represents a highly novel, innovative and promising therapeutic intervention with high potential to alleviate neuropathological change associated with common, progressive and incapacitating neurological disorders of the human CNS.
